# Predictive nomogram model for severe coronary artery calcification in end-stage kidney disease patients

**DOI:** 10.1080/0886022X.2024.2365393

**Published:** 2024-06-14

**Authors:** Xinfang Tang, Hanyang Qian, Shijiu Lu, Hui Huang, Jing Wang, Fan Li, Anning Bian, Xiaoxue Ye, Guang Yang, Kefan Ma, Changying Xing, Yi Xu, Ming Zeng, Ningning Wang

**Affiliations:** a Department of Nephrology, the First Affiliated Hospital of Nanjing Medical University, Jiangsu Province Hospital, Nanjing, China; b Department of Nephrology, the Affiliated Lianyungang Oriental Hospital of Kangda College of Nanjing Medical University, Lianyungang, China; c Department of Nephrology, Nanjing Tongren Hospital, Nanjing, China; d Center for Medical Big Data, Nanjing Drum Tower Hospital, Affiliated Drum Tower Hospital, Medical School of Nanjing University, Nanjing, China; e Department of Nephrology, Nanjing BenQ Medical Center, Nanjing, China; f Department of Critical Medicine, Geriatric Hospital of Nanjing Medical University, Nanjing, China; g Department of Imaging, the First Affiliated Hospital of Nanjing Medical University, Jiangsu Province Hospital, Nanjing, China

**Keywords:** End-stage kidney disease, coronary artery calcification, Agatston coronary artery calcification score, left anterior descending artery, chronic kidney disease-mineral and bone disorders, nomogram

## Abstract

**Introduction:**

The Agatston coronary artery calcification score (CACS) is an assessment index for coronary artery calcification (CAC). This study aims to explore the characteristics of CAC in end-stage kidney disease (ESKD) patients and establish a predictive model to assess the risk of severe CAC in patients.

**Methods:**

CACS of ESKD patients was assessed using an electrocardiogram-gated coronary computed tomography (CT) scan with the Agatston scoring method. A predictive nomogram model was established based on stepwise regression. An independent validation cohort comprised of patients with ESKD from multicentres.

**Results:**

369 ESKD patients were enrolled in the training set, and 127 patients were included in the validation set. In the training set, the patients were divided into three subgroups: no calcification (CACS = 0, *n* = 98), mild calcification (0 < CACS ≤ 400, *n* = 141) and severe calcification (CACS > 400, *n* = 130). Among the four coronary branches, the left anterior descending branch (LAD) accounted for the highest proportion of calcification. Stepwise regression analysis showed that age, dialysis vintage, β-receptor blocker, calcium-phosphorus product (Ca × P), and alkaline phosphatase (ALP) level were independent risk factors for severe CAC. A nomogram that predicts the risk of severe CAC in ESKD patients has been internally and externally validated, demonstrating high sensitivity and specificity.

**Conclusion:**

CAC is both prevalent and severe in ESKD patients. In the four branches of the coronary arteries, LAD calcification is the most common. Our validated nomogram model, based on clinical risk factors, can help predict the risk of severe coronary calcification in ESKD patients who cannot undergo coronary CT analysis.

## 1. Introduction

The risks of death in chronic kidney disease (CKD) patients are eight times higher than that of the general population, and cardiovascular disease (CVD) accounts for more than 50% of the causes [1]. As an important component of vascular calcification (VC), coronary artery calcification (CAC) is closely related to CVD and high mortality in patients with CKD [2]. In a study of 1,579 participants with CKD G1-G5 without kidney replacement therapy, the results show that the coronary artery calcification score(CACS) was independently associated with adverse cardiovascular outcomes and all-cause death in patients with CKD [3]. Patients with severe coronary calcification whose CACS is greater than 400, have an extremely high atherosclerotic plaque load, which increases their cardiovascular risk [4]. All-cause mortality and cardiovascular events were significantly higher in peritoneal dialysis (PD) patients with a CACS > 400 than in those with a CACS = 0 [5].

In the general population, CACS is evaluated using computed tomography (CT) scans. The Agatston score is valuable for the prognosis of cardiovascular disease [6]. The factors influencing the occurrence and development of CACS include age, body mass index (BMI), diabetes, hypertension, smoking, inflammatory factors, fibrinogen, and dyslipidemia, etc [7–11]. Intervention studies on CAC mainly focus on controlling lipid abnormalities [12,13]. In CKD patients, in addition to the above-mentioned influencing factors, several unique pathophysiological characteristics have been observed. These include decreased glomerular filtration rate, hyperphosphatemia, a high calcium-phosphorus product, secondary hyperparathyroidism (SHPT), oxidative stress, systemic inflammation, protein-energy malnutrition, asymmetric dimethylarginine (ADMA), p-cresol, and Fetuin-A. These factors have been proven to be associated with CAC [5,14–19]. Although CT scanning is a noninvasive imaging method used for detecting CACS, its availablity in primary hospitals is limited.

In this study, we conducted a retrospective analysis of the characteristics of CAC in ESKD patients, using the Agatston CACS. We examined the incidence and clinical features of severe total coronary calcification, defined as a CACS greater than 400, in ESKD patients. The primary objective of this study was to develop a nomogram model that can predict the presence of severe CAC in ESKD patients. The proposed model aims to provide valuable insights for the prevention and treatment of CAC, particularly in primary care settings.

## 2. Materials and methods

### 2.1. Study populations

This retrospective study included 369 CKD5 patients who received treatment at the Department of Nephrology, the First Affiliated Hospital of Nanjing Medical University, from June 2017 to April 2022. The patients in this group constituted the training set. The inclusion criteria were as follows [20]: (1) individuals aged between 18 and 75 years old; (2) eGFR (estimated using the CKD-EPI formula) less than 15 mL/min/1.73 m^2^; and (3) individuals who had undergone predialysis or regular dialysis for a duration of three or more months.

Exclusion criteria included: (1) Estimated survival time < 6 months; (2) Fever or infection; (3) Pregnant or lactating women; (4) Severe liver disease, chronic obstructive pulmonary disease, malignant tumor or serious mental disease; (5) History of severe congenital heart disease, atrial fibrillation, atrial flutter, high-grade atrioventricular block and permanent pacemaker implantation [21]; (6) Arterial calcification cannot be detected or the results are unreliable, such as arrhythmia, cardiac stent implantation, amputation or serious peripheral vascular diseases [22]; (7) History of other concomitant diseases affecting calcium status in the body and soft tissue calcification [23], such as sarcomatoid nodules, multiple myeloma, human immunodeficiency virus (HIV, AIDS virus), amyloidosis and primary parathyroid disease.

We refer to the published work [5,24]. The patients were divided into three subgroups according to the overall degree of CACS: no calcification (CACS = 0), mild calcification (0 < CACS ≤ 400), and severe calcification (CACS > 400).

Additional 127 patients diagnosed with CKD5, who received treatment at Department of Nephrology, the First Affiliated Hospital of Nanjing Medical University and the Affiliated Lianyungang Oriental Hospital of Kangda College of Nanjing Medical University from May 2022 to May 2023, were included as the validation cohort. These patients were selected based on the same criteria as the initial cohort, with the aim of validating the predictive effectiveness of the nomogram model.

### 2.2. Measurements and assessments

The general information, baseline laboratory examinations, and CACS evaluated using CT scan were documented for all participants.This study was approved by the ethics committee of the First Affiliated Hospital of Nanjing Medical University (ethics No.: 2011-sr-072. 2019-sr-368). All participants have signed informed consent.

#### 2.2.1. Research process

All venous blood samples were collected from participants at 7 am after an overnight fast. These samples were then analyzed for routine blood tests, biochemical indices such as calcium (Ca) and phosphorus (P), bone alkaline phosphatase (BAP), 25 hydroxyvitamin D (25-OH-D), and intact parathyroid hormone (iPTH). For hemodialysis (HD) patients, blood samples were obtained prior to dialysis. The assessment of CACS using CT scan must be done within one week of the hematological examination.

#### 2.2.2. Laboratory examinations

The routine blood tests were detected by LH-750 blood cell analyzer (Beckman Coulter, Fullerton, CA, USA). Automatic biochemical analyzer (AU5400; Olympus Corporation, Tokyo, Japan) was used to detect blood biochemical indices. The serum iPTH level was detected by immunoassay system (Unicel Dxi800 Access; Beckman Coulter, Fullerton, CA, USA). The serum BAP level was determined by enzyme-linked immunosorbent assay (ELISA) and serum 25-OH-D level was determined by radioimmunoassay. The correction formula of blood calcium: serum corrected calcium (mmol/l)=total serum calcium (mmol/l)+ [40-blood albumin (g/l)] × 0.02.

#### 2.2.3. CACS evaluation

Continuous sections were obtained without gaps through an electrocardiogram (ECG)-gated coronary CT plain scan with the Agatston scoring method. The scan range was from the tracheal carina to 1 cm below the heart apex. All sections were evaluated to determine the presence and number of coronary calcifications. The threshold of calcification was set as a CT density of 130 Hounsfield units (Hu) with an area ≥1 mm^2^. All pixels with a CT density ≥ 130 Hu were displayed in each section. A “region of interest” was defined around all the calcifications found in the coronary artery to measure the calcification area in mm^2^. The density score was determined based on the maximum CT attenuations in each region of interest automatically: 1 (score) = 130 to 199 HU, 2 = 200 to 299 HU, 3 = 300–399 HU, 4 = greater than or equal to 400 HU. The score of each region of interest is calculated by multiplying the density score and the region’s area. The total CACS was determined by adding the scores of all sections.

### 2.3. Statistical analysis

The continuous variables are presented as mean ± standard deviation or median (Q1, Q3), and the categorical variables are presented as number (percentage). Baseline characteristics between groups were compared using the unpaired, two-tailed t test or Mann-Whitney test for continuous variables, depending on the data distribution, and the *χ*^2^ test was used for categorical variables.

Univariate logistic regression analysis was used to screen the factors affecting severe LAD calcification (LAD CACS > 183.4) and severe total CACS (total CACS > 400), including the baseline characteristics and laboratory indicators. The results were presented by odds ratio (OR) and 95% confidence interval (CI). Multivariate logistic stepwise regression analysis was used to analyze the factors related to severe total CACS.

According to the results of multiple stepwise regression analysis, a nomogram model was established to predict the risk of severe CACS in ESKD patients, and used the area under the curve (AUC) value of the receiver operating characteristic (ROC) curve to evaluate the predictive performance of the model.

All statistical analyses were performed using R foundation for Statistical Computing (version 4.3.1), unless otherwise specified. A *p-*value of <0.05 was considered statistically significant.

## 3. Results

### 3.1. General information of the study population

A total of 369 ESKD patients were enrolled, including predialysis patients (13.01%), hemodialysis (HD) patients (64.23%), and PD patients (23.76%). The average age of participants was 49.41 ± 12.14 years old, and the average dialysis vintage was 73.00 (24.00–120.00) months. The drugs used to treat chronic kidney disease-mineral and bone disorders (CKD-MBD) include phosphorus binders (39.57%), active vitamin D sterols (35.50%), and cinacalcet (27.10%).

The patients were divided into three subgroups according to the overall degree of CACS: no calcification (CACS = 0, *n* = 98), mild calcification (0 < CACS ≤ 400, *n* = 141), and severe calcification (CACS > 400, *n* = 130). Age, BMI, diastolic blood pressure, dialysis mode, dialysis vintage, whether complicated with hypertension, and the use of β-receptor blockers were significantly different among the subgroups (*p* < 0.05). Among these, HD patients (43.46%) are more likely to have severe CAC compared to PD patients (22.36%). The serum hemoglobin (Hb), hematocrit (HCT), total cholesterol (TC), low density lipoprotein cholesterol (LDL-C), high density lipoprotein cholesterol (HDL-C), Ca, corrected Ca, Ca × P, log(ALP), log(BAP), and log(iPTH) also showed significantly different (*p* < 0.05) (Table 1).

**Table 1.** Clinical characteristics and laboratory results of ESKD patients subgrouped by CACS.

Variables	CACS = 0 (*n* = 98)	0 < CACS ≤ 400 (*n* = 141)	CACS > 400 (*n* = 130)	*p*
Demographics				
Age (years)	42.28 ± 11.47	50.88 ± 11.46	53.18 ± 11.12	<0.001*
Women, *n* (%)	44.00(44.90)	61.00(43.26)	49.00(37.69)	0.260
BMI (kg/m^2^)	21.45 ± 3.54	23.04 ± 3.76	22.76 ± 3.92	0.018*
SBP (mmHg)	140.65 ± 23.67	137.88 ± 19.80	138.79 ± 25.40	0.587
DBP (mmHg)	88.27 ± 15.52	82.37 ± 13.55	82.26 ± 14.31	0.003*
Dialysis mode, *n* (%)				
Predialysis	18.00(18.37)	18.00(12.77)	8.00(6.15)	0.004*
Hemodialysis	43.00(43.88)	91.00(64.54)	103.00(79.23)	<0.001*
Peritoneal dialysis	33.00(33.67)	33.00(23.74)	19.00(14.62)	<0.001*
Dialysis vintage(months)	42.00(3.00–96.00)	72.00(24.00–108.00)	96.00(60.00–132.75)	<0.001*
Comorbidities, *n* (%)				
Diabetic mellitus	9.00(9.18)	26.00(18.44)	24.00(18.46)	0.073
Hypertension	69.00(70.41)	106.00(75.18)	112.00(86.15)	0.004*
Cause of ESKD, *n* (%)				
CGN	72.00(73.47)	93.00(65.96)	90.00(69.23)	0.554
DN	7.00(7.14)	17.00(12.06)	14.00(10.77)	0.418
HN	2.00(2.04)	5.00(3.55)	2.00(1.54)	0.737
Polycystic kidney disease	4.00(4.08)	6.00(4.26)	8.00(6.25)	0.435
Other	13.00(13.27)	20.00(14.18)	17.00(13.08)	0.948
Medication history, *n* (%)				
Lipid-lowering treatment	8.00(8.25)	18.00(12.77)	17.00(13.18)	0.276
Dihydropyridine CCBs	49.00(50.52)	71.00(50.35)	74.00(57.36)	0.280
ACEI/ARB	33.00(34.02)	32.00(22.70)	46.00(35.94)	0.601
β-receptor blocker	35.00(36.08)	54.00(38.30)	64.00(49.61)	0.034*
Phosphate binders	38.00(39.18)	46.00(32.62)	62.00(48.06)	0.124
Active vitamin D sterols	33.00(34.02)	56.00(39.72)	42.00(32.56)	0.736
Cinacalcet	22.00(22.68)	37.00(26.24)	41.00(31.78)	0.123
Laboratory values				
Hemoglobin (g/L)	96.97 ± 20.51	102.23 ± 18.37	103.78 ± 21.46	0.014*
Hematocrit (%)	29.76 ± 6.58	31.84 ± 5.92	32.39 ± 6.64	0.003*
Glucose (mmol/L)	4.41 ± 1.17	4.69 ± 2.07	4.70 ± 2.35	0.294
Creatinine (μmol/L)	918.88 ± 336.59	882.82 ± 299.19	886.81 ± 285.46	0.462
Urea (mmol/L)	24.33 ± 9.87	23.77 ± 11.56	21.91 ± 7.09	0.055
TC (mmol/L)	4.40 ± 1.54	4.22 ± 1.04	3.92 ± 1.06	0.003*
TG (mmol/L)	1.76 ± 1.31	1.72 ± 1.10	1.76 ± 1.32	0.991
LDL-C (mmol/L)	2.77 ± 1.14	2.63 ± 0.77	2.44 ± 0.81	0.006*
HDL-C (mmol/L)	1.08 ± 0.34	1.01 ± 0.28	0.92 ± 0.25	<0.001*
Lpa (mmol/L)	374.87 ± 279.48	331.27 ± 280.60	302.00 ± 282.33	0.055
Albumin (g/L)	37.03 ± 5.36	37.57 ± 5.66	36.85 ± 5.01	0.724
Ca (mmol/L)	2.28 ± 0.26	2.34 ± 0.26	2.42 ± 0.26	<0.001*
Adjusted Ca (mmol/L)	2.35 ± 0.23	2.39 ± 0.23	2.48 ± 0.23	<0.001*
Phosphorus (mmol/l)	1.98 ± 0.47	2.03 ± 0.51	2.16 ± 0.54	0.009*
Ca × P (mmol^2^/L^2^)	4.66 ± 1.18	4.89 ± 1.39	5.37 ± 1.47	<0.001*
ALP (U/L)	105.40(75.25–195.80)	134.90(83.00–255.00)	204.50(105.87–348.23)	0.255
Log(ALP)	4.95 ± 0.96	5.07 ± 0.86	5.35 ± 0.90	<0.001*
BAP (μg/L)	26.19 ± 27.63	31.41 ± 27.54	45.38 ± 38.34	<0.001*
Log(BAP)	2.81 ± 1.12	3.15 ± 0.75	3.42 ± 0.93	<0.001*
25-OH-D (ng/dL)	40.40 ± 29.52	44.74 ± 28.54	44.51 ± 24.08	0.301
iPTH (pg/ml)	444.90(169.20–1263.70)	795.25(249.40–1321.4)	1205.30(371.95–1903.10)	<0.001*
Log(iPTH)	6.05 ± 1.25	6.33 ± 1.25	6.64 ± 1.22	<0.001*

*Abbreviations*. ESKD: end-stage kidney disease; BMI: body mass index; SBP: systolic blood pressure; DBP: diastolic blood pressure; CGN: chronic glomerulonephritis; DN: diabetic nephropathy; HN: hypertensive nephropathy; CCB: calcium channel blocker; ACEI/ARB: angiotensin-converting enzyme inhibitors/angiotensin II receptor blockers; TC: total cholesterol; TG: triglyceride; LDL-C: low-density lipoprotein cholesterol; HDL-C: high-density lipoprotein cholesterol; Lpa: lipoprotein a; Ca: calcium; P: phosphorus; ALP: alkaline phosphatase; BAP: bone-type alkaline phosphatase; 25-OH-D: 25 hydroxyvitamin D; iPTH: intact parathyroid hormone. *p*-Values are obtained from comparisons between the three groups, the bold values indicated by an asterisk (*) mean* p* < 0.05. Data forms are expressed as mean ± standard deviation or median (Q1–Q3), except where indicated.

### 3.2. CACS is significantly higher in ESKD patients undergoing HD

**Figure 1.**
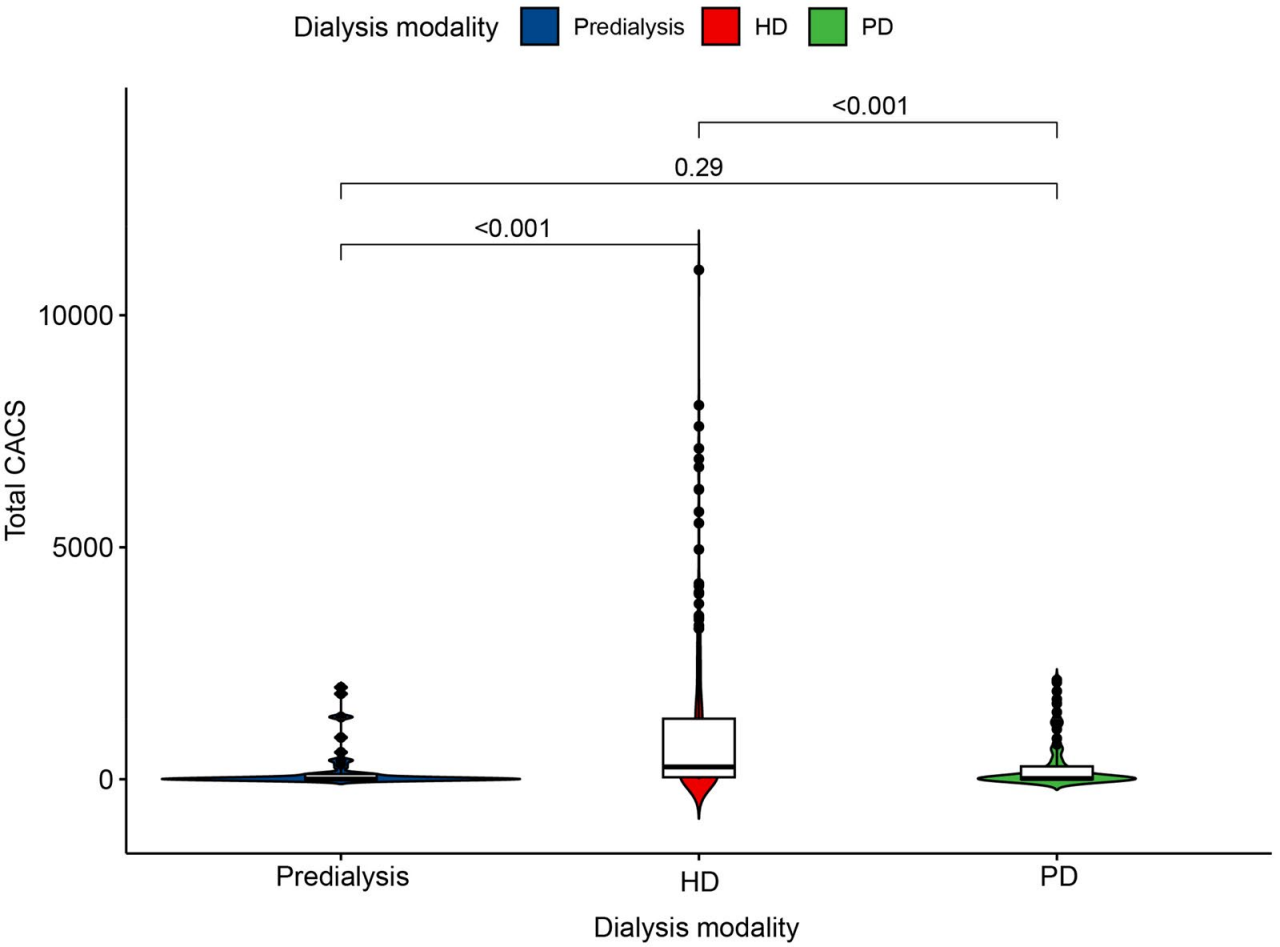
Comparison of CAC in ESKD patients with different dialysis modalities. Abbreviations: ESKD: end-stage kidney disease; CAC: coronary artery calcification; HD: hemodialysis; PD: peritoneal dialysis.

We conducted an observation of CAC in different dialysis modalities (Figure 1). Depending on the dialysis modality, we divided the 369 patients into three groups: the predialysis group (*n* = 44), the HD group (*n* = 237), and the PD group (*n* = 85). The CACS was found to be significantly higher in the HD group compared to the predialysis group (*p* < 0.001). Furthermore, the CACS remained persistently higher in the HD group than in the PD group (*p* < 0.001).

### 3.3. The percentage of LAD calcification was highest among the four coronary branches in ESKD patients

**Figure 2.**
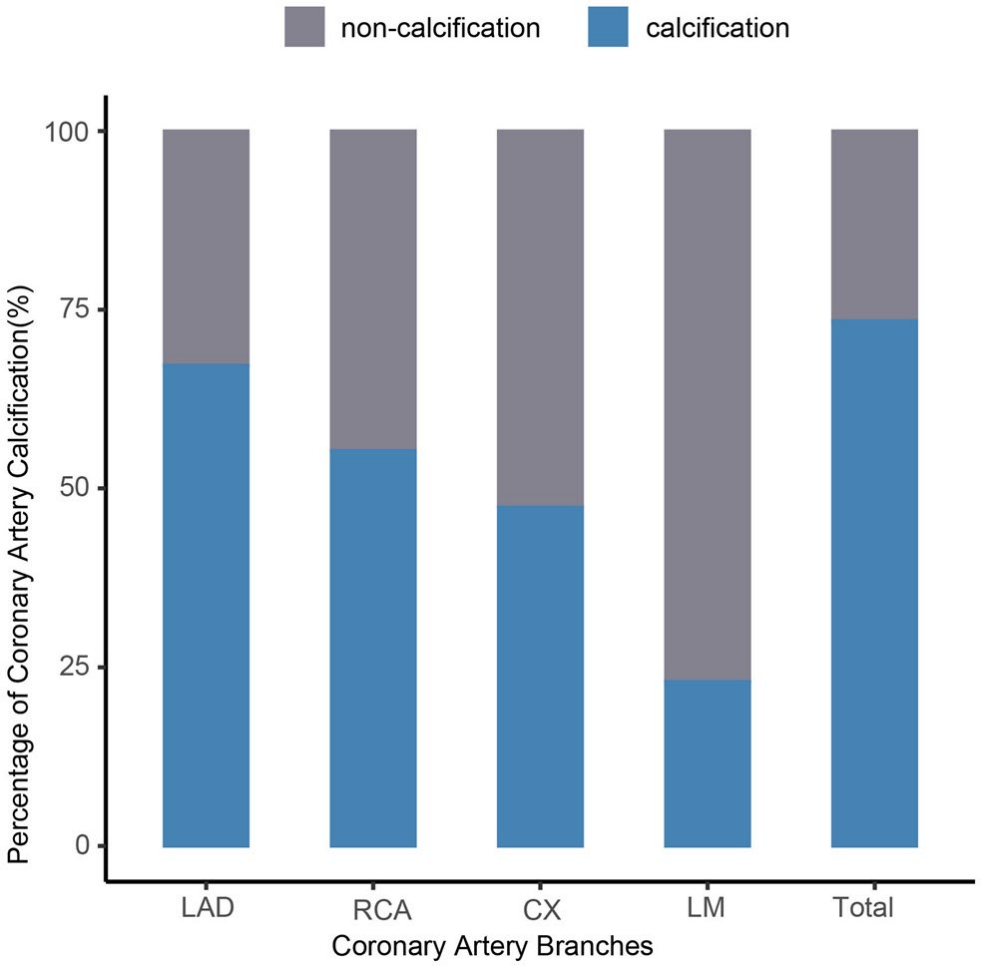
Percentage of CAC in the four coronary branches. Abbreviations: LAD: left anterior descending branch; LM: left main trunk; CX: circumflex branch; RCA: right coronary artery; CAC: coronary artery calcification.

We conducted a percentage analysis of coronary artery calcification (CACS > 0) in the four coronary branches of ESKD patients (Figure 2). Out of the 369 ESKD patients, 248 individuals (67.21%) exhibited calcification in LAD, 85 individuals (23.04%) exhibited calcification in the left main trunk (LM) calcification, 175 individuals (47.42%) exhibited calcification in the circumflex branch (CX), and 204 individuals (55.28%) exhibited calcification in the right coronary artery (RCA) (*p* < 0.05). The percentage of LAD calcification was highest among the four coronary branches in ESKD patients. We analyzed the calcification of the four coronary branches in the pre-dialysis, HD, and PD groups, respectively, and found that the percentage of LAD calcification remained the highest in all of them (supplemental Figure S1, Table S1).

### 3.4. Imaging characteristics of ESKD patients with varying degrees of CAC

**Figure 3.**
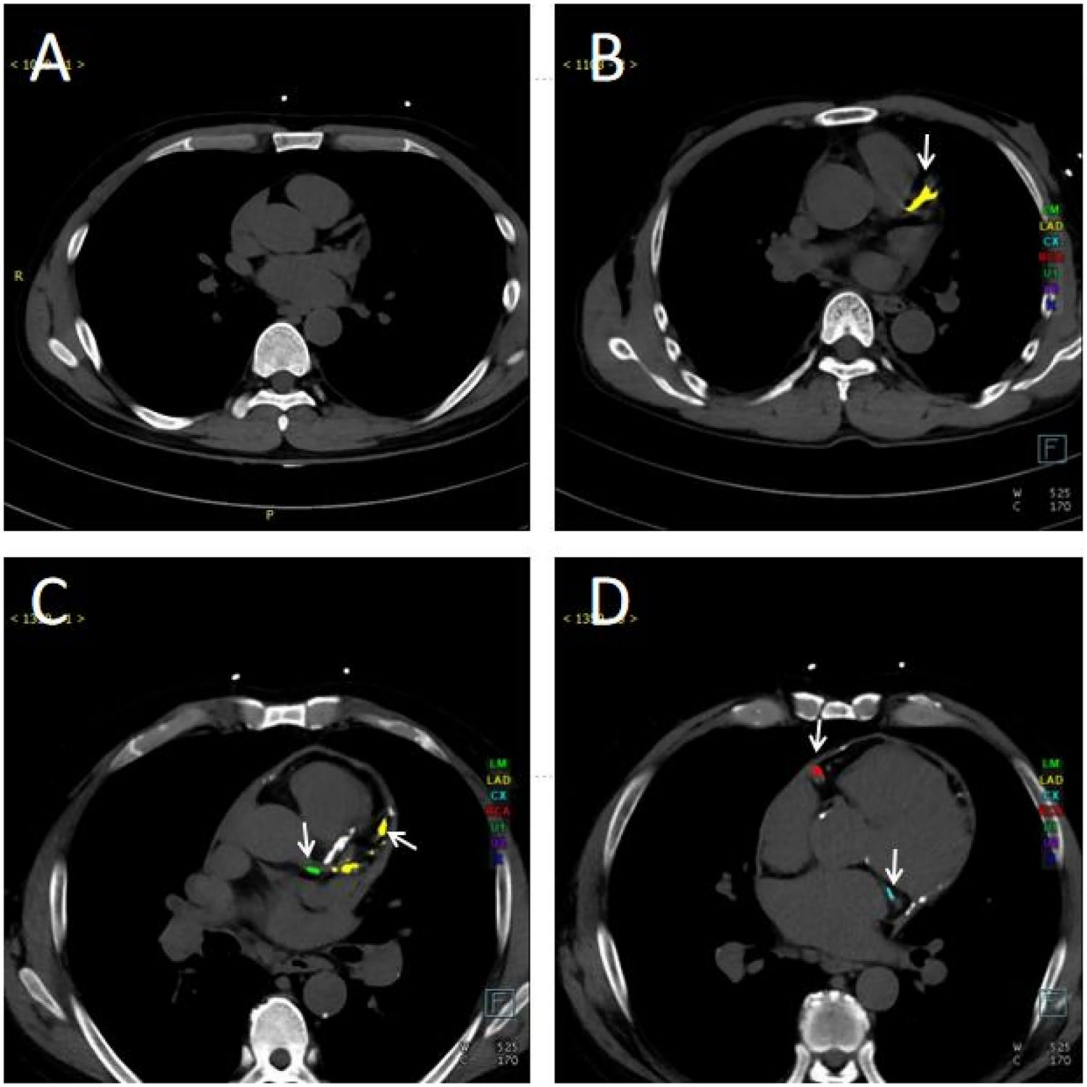
Cross-sectional CT images of ESKD patients with varying degrees of CACS. (A) An ESKD patient without coronary calcification. (B) An ESKD patient with calcification only in the LAD. (C–D) An ESKD patient with calcification in all four coronary branches. (C) Calcification in the left main trunk and left anterior descending branch. (D) Calcification in the circumflex branch and right coronary artery. Different colors represent each coronary branch with calcification (green: left main trunk; yellow: left anterior descending branch; blue: circumflex branch; red: right coronary artery), and the arrows indicated the calcified branches. Abbreviations: CT: computed tomography; ESKD: end-stage kidney disease; LAD: left anterior descending branch.

Figure 3 depicts CT images of patients exhibiting different degrees of CAC. Specifically, Figure 3(A) illustrates a patient who does not present coronary calcification (calcification score: 0). In contrast, Figure 3(B) displays a patient with sole calcification in the LAD (calcification score: LAD = 647.9). Figure 3(C) (calcification score: LM = 156.5, LAD = 2320.7) and Figure 3(D) (calcification score: CX = 2317.5, RCA = 2329.3) show calcification of 4 coronary branches in one patient.

### 3.5. Clinical characteristics of LAD calcification in ESKD patients

A total of 369 patients were categorized into three subgroups based on their LAD calcification score tertiles namely: low (score of 0), intermediate (score ranging from 0 to 183.4), and high (score exceeding 183.4) (supplemental Table S2). The clinical characteristics and laboratory results of these subgroups were observed and analyzed. Statistical analysis revealed significant differences in age, BMI, systolic blood pressure, dialysis mode, dialysis vintage, and the presence of concomitant hypertension among the three subgroups (*p* < 0.05). There were statistically significant differences observed in the laboratory indicators between the subgroups, including Hb, HCT, TC, HDL-C, LDL-C, Ca, adjusted Ca, P, Ca × P, log(ALP), log(BAP), and log(iPTH) (*p* < 0.05).

### 3.6. Risk factors associated with severe CAC in patients with ESKD

We examined the risk factors associated with severe calcification, as indicated by a LAD coronary artery calcium score (CACS) exceeding 183.4, as well as the overall CACS exceeding 400 (supplemental Table S3). Through univariate analysis, we observed that age, dialysis mode, dialysis vintage, and the use of β-blockers demonstrated significant associations with the occurrence of severe CAC in ESKD patients (*p* < 0.05). Patients with HD (OR, 2.989; 95% CI, 1.823–4.903; *p* < 0.001) were found to have a higher propensity for developing severe CAC compared to patients with PD (OR, 0.444; 95% CI, 0.253–0.779; *p* < 0.05). Blood lipid metabolism-related indicators, specifically TC, LDL-C, and HDL-C levels, as well as blood bone metabolic indexes, including Ca, adjusted Ca, P, Ca × P, log(ALP), log(BAP) and log(iPTH) levels, were observed to have a statistically significant association with the risk of severe CAC (*p* < 0.05). The risk factors related to severe calcification of the LAD showed similar consistency with those associated with total CACS, with the exception of concomitant hypertension and the use of oral phosphorus binders, which were not found to be linked to severe LAD calcification.

### 3.7. Stepwise regression analysis of risk factors associated with severe CAC in patients with ESKD

A stepwise regression analysis was conducted to identify the risk factors associated with severe CAC in patients with ESKD (Table 2). After adjusting for confounding variables, three independent risk factors for severe CAC in ESKD patients were identified: age (OR, 1.071; 95% CI, 1.037–1.106; *p* < 0.001), dialysis vintage (OR,1.008; 95% CI, 1.001–1.015; *p* = 0.028), and the use of oral β-receptor blocker (OR, 2.309; 95% CI, 1.095–4.870; *p* = 0.028). Among the blood bone metabolic indexes examined, only Ca × P (OR, 1.333; 95% CI, 1.007–1.771; *p* = 0.045) and log(ALP) (OR, 3.026; 95% CI, 1.080–8.484; *p* = 0.035) levels exhibited a significant association with the occurrence of severe CAC.

**Table 2.** Stepwise regression analysis of clinical data and severe total CACS in patients with ESKD.

Variables	OR (95%CI)	*p*
Age	1.071 (1.037,1.106)	<0.001*
BMI	0.992 (0.891,1.103)	0.879
SBP	1.010 (0.995,1.026)	0.190
Dialysis vintage	1.008 (1.001,1.015)	0.028*
Diabetic mellitus	2.324 (0.870,6.208)	0.093
Lipid-lowering treatment	0.981 (0.356,2.703)	0.970
ACEI/ARB	1.654 (0.767,3.563)	0.199
β-receptor blocker	2.309 (1.095,4.870)	0.028*
Cinacalcet	0.456 (0.188,1.103)	0.081
TC	0.582 (0.119,2.855)	0.505
TG	0.924 (0.599,1.427)	0.722
LDL-C	2.455 (0.379,15.916)	0.346
HDL-C	0.816 (0.085,7.860)	0.860
Ca × P	1.335 (1.007,1.771)	0.045*
Log(ALP)	3.026 (1.080,8.484)	0.035*
Log(BAP)	0.928 (0.399,2.157)	0.861

Abbreviations: ESKD: end-stage kidney disease; BMI: =body mass index; SBP: systolic blood pressure; ACEI/ARB: angiotensin-converting enzyme inhibitors/angiotensin II receptor blockers; TC: total cholesterol; TG: triglycerides; LDL-C: low-density lipoprotein cholesterol; HDL-C: high-density lipoprotein cholesterol; Ca × P: calcium-phosphorus product; ALP: alkaline phosphatase; BAP: bone-type alkaline. The *p*-values in the table were obtained through logistic stepwise regression analysis of each index and calcification score. The bold values indicated by an asterisk (*) mean *p* < 0.05.

### 3.8. Nomogram model based on clinical data for predicting the risks of severe CAC in ESKD patients and internal validation

**Figure 4.**
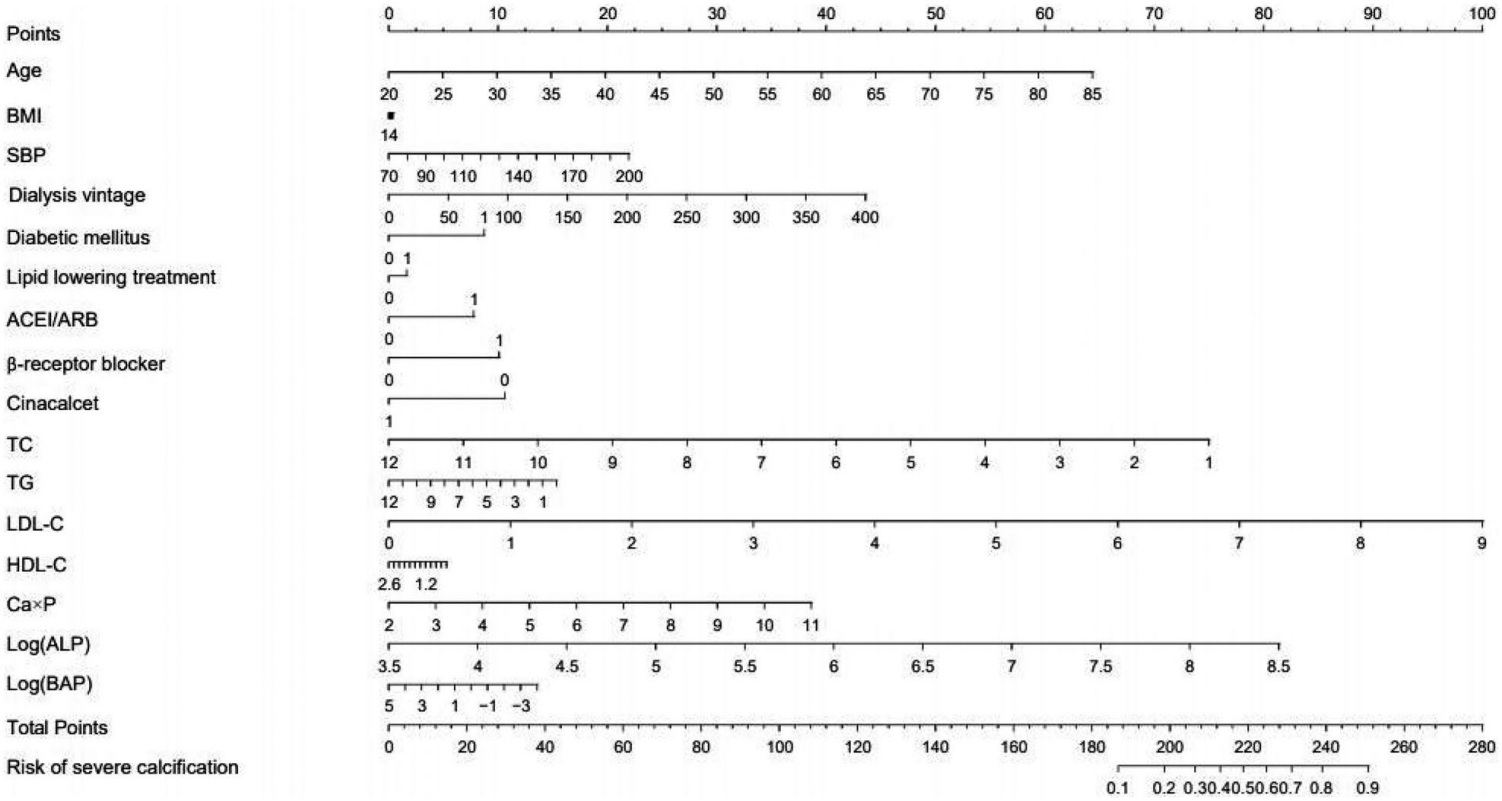
Nomogram model to predict the risks of severe CAC based on clinical data of ESKD patients. Based on the value assigned to each patient indicator (0 or 1 for diabetes history and medication history), a dot was plotted on the horizontal line corresponding to the respective item. A vertical line was then drawn upwards to intersect the horizontal line representing the score, thus determining the specific score for that particular item. The total score was calculated by adding up the scores from each item. A dot was plotted on the horizontal line representing the total score. A vertical line was then drawn downwards to intersect the horizontal line corresponding to the risk of severe coronary calcification, giving us the specific value for calcification risk.

In order to predict the risks of severe CAC in ESKD patients, a nomogram model was established. This model takes into account various clinical factors, including age, BMI, SBP, dialysis vintage, history of diabetes, medication history, and laboratory indicators such as TC, triglyceride (TG), LDL-C, HDL-C, Ca × P, log(ALP), and log(BAP) (Figure 4).

**Figure 5.**
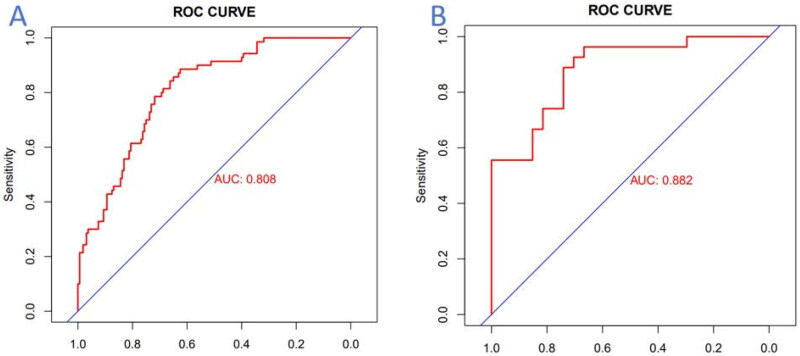
ROC curves of nomogram model. (A) ROC curve of the nomogram in the training cohort; (B) ROC curve of the nomogram in the validation cohort. *Note.* The red curve represents the model’s performance in predicting the risks of severe coronary calcification, with the horizontal axis representing specificity and the vertical axis representing sensitivity. *Abbreviations*. AUC: area under curve; ROC: receiver operating characteristic.

The nomogram model’s predictive performance was internally validated using AUC analysis of the ROC curve (Figure 5(A)). The AUC of this model was determined to be 0.808 (95% CI: 0.751*–*0.866). Additionally, the sensitivity and specificity were calculated to be 0.843 (95% CI: 0.758*–*0.928) and 0.701 (95% CI: 0.629*–*0.772), respectively. These findings suggest that the histogram based on clinical data exhibits a reliable predictive ability in estimating the risks of severe CAC in ESKD patients, demonstrated by its high sensitivity and specificity.

### 3.9. Independent validation of the nomogram

A validation set consisting of 128 patients with stage 5 CKD were utilized for independent validation of the nomogram. This set was categorized into three subgroups based on their coronary calcification levels: no calcification (*n* = 22, 17.32%), mild calcification (*n* = 51, 40.15%), and severe calcification (*n* = 54, 42.52%). Among the patients, 80 (63.00%) were males and 47 (37.00%) were females. The mean age of the patients was 52.41 ± 14.34 years, with a mean duration of dialysis of 60.00 (24.00–124.00) months. The average BMI was 23.10 ± 3.89 kg/m^2^. In terms of comorbidities, 19.69% of the patients with ESKD had diabetes mellitus, and 81.10% of the patients had hypertension (supplemental Table S4).

The predictive model underwent external validation through AUC analysis of the ROC curve, utilizing an independent validation dataset (Figure 5(B)). The model achieved an AUC score of 0.882 (95% CI: 0.794–0.970), with a sensitivity of 0.926 (95% CI: 0.827–1.000) and a specificity of 0.704 (95% CI: 0.531–0.876). These findings suggest that the predictive model demonstrates commendable predictive capabilities.

## 4. Discussion

In the Multi-Ethnic Study of Atherosclerosis (MESA) project [25], 684 participants with a baseline eGFR < 60 mL/min/1.73 m^2^ had a 66% incidence rate of CAC. Przemyslaw reported that the proportion of CAC in HD patients was 73.1%, which was higher than that in the control group (35.7%) [19]. After following up with 179 PD patients for 30.6 ± 16.2 months and 104 HD patients for 43.8 ± 19.3 months [26], CACS was proven to be an independent predictor of all-cause mortality, CVD, and cardiovascular mortality in ESKD patients. In our study, 73.44% of ESKD patients had CAC, and 35.23% had severe calcification (CACS > 400). Previous studies have reported that the severity of CAC is more significant in ESKD patients with a longer duration of dialysis [17,27], which aligns with our findings. We are the first to reveal that patients with HD are more prone to developing severe CAC compared to those with PD.

CACS in CKD patients has been found to correlate with various traditional cardiovascular risk factors, including age, gender, BMI, SBP, diabetes, and use of antihypertensive medications [14,27,28]. In our study, we observed differences in age, BMI, DBP, and hypertension among subgroups stratified based on the severity of CAC. Upon conducting a multivariate regression analysis, age emerged as an independent risk factor for severe CAC, which is consistent with previous research conducted at our center [29].

We discovered that the highest rate of calcification was observed in the LAD branch. Among the four main coronary branches, the LAD is the most commonly affected vessel in cardiovascular disease [30], and exhibits the highest proportion of calcification, accounting for 67.21%. Blanke H et al. [31] found that the most frequent infarct-related artery among patients admitted with chest pain or discharged from the hospital after an acute myocardial infarction is the LAD (44–56% of cases), followed by the RCA (27–39%) and the CX (17%). CAC is likely to be an important factor. We have demonstrated that the factors influencing LAD calcification are comparable to those affecting the total CACS.

Our study revealed that levels of serum-corrected calcium and phosphorus were identified as risk factors for severe CAC. Elevated serum calcium levels were found to directly influence the progression of CAC [32]. Furthermore, hyperphosphatemia was shown to be positively associated with CAC in patients with CKD [21]. Consistently strict phosphate control may slow the progression of coronary and valvular calcifications in incident patients undergoing hemodialysis [33]. In our study, we found that the Ca × P product was an independent risk factor for severe CAC. However, blood calcium, phosphorus, and iPTH levels are not independent risk factors for severe CAC, which is consistent with the study by Malluche HH et al. [27]. Additionally, elevated levels of ALP were found to promote vascular calcification, and a positive correlation was observed between high ALP levels and CACS [34]. In a previous study, we also observed a significant association between serum ALP levels and cardiac valve calcification in maintenance HD patients [35]. In line with these findings, we discovered that the ALP served as an independent risk factor for severe CAC. BAP, known as the most important marker of osteoblast differentiation, was reported to be a risk factor for coronary calcification in male HD patients [36]. Finally, serum iPTH levels were positively correlated with CACS [37], confirming our own finding.

In our study, there was no discernible association between oral cinacalcet, serum 25-OH-D levels, and severe coronary artery calcification. Previous research suggests that Vitamin D deficiency is linked to considerable vascular calcification in CKD patients [38]. Nevertheless, a study involving older African American women revealed no significant relationship between abdominal aorta and serum 25-OH-D levels [39]. A large cross-sectional study found a U-shaped relationship between serum 25(OH)D concentration and the risk of abdominal aortic calcifcation(AAC) and severe AAC [40]. Although it has been established that the combination of cinacalcet and low-dose vitamin D can alleviate CAC [16], another prospective study involving HD patients with SHPT [41] found no significant difference between the effect of cinacalcet combined with standard therapy versus standard therapy alone on vascular calcification.

In this study, we find the association of oral β-receptor blockers with the development of severe CAC. Past research has established the presence of β2-adrenergic receptor (AR) on the surface of human osteoblasts (OBs), and it has been suggested that β2-AR agonists could potentially hinder the proliferation of OBs [42]. Wu et al. [43] discovered that the β-receptor blocker propranolol increased the expression levels of osteogenesis-associated genes in New Zealand rabbits, such as bone morphogenetic protein (BMP2), RUNX family transcription factor (RunX2), collagen (COL-1), and osteocalcin (OCN). Consequently, this augmentation promotes the osteogenic differentiation of mesenchymal stem cells (MScs) and OBs. Vascular calcification is a gene-regulated biological process similar to bone mineralization, involving osteogenic differentiation [44]. However, further investigations are necessary to better understand the effects of β-receptor blockers on CAC.

The TC concentration has been proven to be independently associated with the incidence rate of CAC. Hypercholesterolemia is considered a risk factor for CAC [45]. There is a positive correlation between LDL-C and CACS [13,46]. LDL-C levels have also been positively correlated with the risk of CAC events, although this association diminishes with increasing HDL-C levels [13]. In contrast, HDL-C has been shown to be negatively correlated with CACS [28]. However, one study reported that HDL-C and its sub-components (HDL2-C and HDL3-C), as well as the HDL2-C/HDL3-C ratio, had no significant relation with the presence or degree of CAC [47]. High TG levels are related to the progression of CAC [46,48]. A cross-sectional study of PD patients found no significant association between CAC and TG, HDL-C, LDL-C [14]. In our study, we aslo found no correlation between lipid metabolism indices and severe CACS.

Here, we developed a predictive nomogram model to predict the risk of CACS> 400 in ESKD patients. The nomogram model takes into account various factors. Both internal and external validation, as indicated by the AUC of the ROC curve, demonstrated the excellent sensitivity and specificity of this nomogram model. Given the challenges associated with conducting CT evaluations for CACS in primary hospitals, the nomogram model serves as a simplified and user-friendly approach that enhances usability in clinical settings.

This risk stratification can aid clinicians in identifying patients who may benefit from more aggressive preventive measures or closer monitoring, making informed decisions regarding whether further Agatston coronary artery calcification score analysis and treatment strategies are needed. On the other hand, the patients at lower risk may not require similar interventions, allowing for a more personalized approach to treatment. Currently, the more definitive drugs for the treatment of vascular calcification are magnesium and sodium thiosulfate. Others are still under clinical investigation [49–51]. The effect of statins on vascular calcification is controversial, with some studies in the general population and animals showing that statins improve vascular calcification, while others indicate they may worsen it or have no effect at all [52]. Most ESKD patients have normal or below-normal serum cholesterol levels and do not benefit from statin therapy, and it may even be detrimental to them [53]. Chen Z et al. found that statin therapy was associated with accelerated CAC progression in CKD patients [54]. Ezetimibe has been shown to significantly prevent atherosclerosis through its lipid-lowering effect [55], but no studies have been conducted on the effect of ezetimibe on VC in CKD. Previous studies have demonstrated that the combination of simvastatin and ezetimibe reduces CAC in CKD patients [56,57]. However, no studies have compared the effects of statins alone versus ezetimibe on vascular calcification in CKD. The nomogram model can also be used to educate and counsel ESKD patients.

However, there are several limitations in our research. This is a retrospective and observational study, lacking relevant bone metabolism markers such as osteopontin, osteoprotegerin, and osteocalcin on haematological examination. Previous studies have shown that bone metabolic markers such as osteopontin, osteoprotegerin, and osteocalcin correlate with vascular calcification [58,59]. Because only Asian patients were examined in this study, the possibility of racial and regional bias cannot be ruled out. CAC progression is a dynamic process, regular reassessment and updating of the risk estimation is necessary for long-term management.

## 5. Conclusion

CAC is both common and severe in ESKD patients. Those on HD patients are more susceptible to severe CAC compared to those on PD. Notably, the percentage of LAD calcification is the highest among the four coronary branches in ESKD patients. Various factors have been identified as independent risk factors for severe CAC, including age, dialysis vintage, use of oral β-receptor blocker, serum Ca × P levels, and ALP levels. Formulating a nomogram model based on clinical data can facilitate the prediction of severe CAC risks in ESKD patients. This can contribute to a reduction in cardiovascular events and mortality rates by improving accurate diagnosis and treatment capability, especially for CKD-MBD patients in primary healthcare settings.

## Supplementary Material

Supplemental Material

## References

[1] Wen Y, Gan H, Li Z, et al. Safety of low-calcium dialysate and its effects on coronary artery calcification in patients undergoing maintenance hemodialysis. Sci Rep. 2018;8(1):1–12. doi:10.1038/s41598-018-24397-w. 29654308 PMC5899126

[2] Elhabashi AF, Sulaibeekh L, Seddiq N, et al. Presepsin level correlates with the development of moderate coronary artery calcifications in hemodialysis patients: a preliminary cross-section design study. Risk Manag Healthc Policy. 2020;13:999–1006. doi:10.2147/RMHP.S262058. 32821182 PMC7422906

[3] Jung C, Yun H, Park JT, et al. Association of coronary artery calcium with adverse cardiovascular outcomes and death in patients with chronic kidney disease: results from the know-ckd. Nephrol Dial Transpl. 2023;38(3):712–721. doi:10.1093/ndt/gfac194. 35689669

[4] Iyer H, Abraham G, Reddy YNV, et al. Risk factors of chronic kidney disease influencing cardiac calcification. Saudi J Kidney Dis Transpl. 2013;24(6):1189–1194. doi:10.4103/1319-2442.121279. 24231482

[5] Xie Q, Ge X, Shang D, et al. Coronary artery calcification score as a predictor of all-cause mortality and cardiovascular outcome in peritoneal dialysis patients. Perit Dial Int. 2016;36(2):163–170. doi:10.3747/pdi.2014.00124. 26224787 PMC4803361

[6] Seitun S, Clemente A, Maffei E, et al. Prognostic value of cardiac ct. Radiol Med. 2020;125(11):1135–1147. doi:10.1007/s11547-020-01285-w. 33047297

[7] Blaha MJ, Budoff MJ, Defilippis AP, et al. Associations between c-reactive protein, coronary artery calcium, and cardiovascular events: implications for the jupiter population from mesa, a population-based cohort study. Lancet (London, England). 2011;378(9792):684–692. doi:10.1016/S0140-6736(11)60784-8. 21856482 PMC3173039

[8] Antonopoulos S, Mylonopoulou M, Angelidi AM, et al. Association of matrix γ-carboxyglutamic acid protein levels with insulin resistance and Lp(a) in diabetes: a cross-sectional study. Diabetes Res Clin Pract. 2017;130:252–257. doi:10.1016/j.diabres.2017.06.015. 28654853

[9] Mathieu P, Boulanger M. Autotaxin and lipoprotein metabolism in calcific aortic valve disease. Front Cardiovasc Med. 2019;6:18. doi:10.3389/fcvm.2019.00018. 30881959 PMC6405425

[10] Ceponiene I, Li D, El KS, et al. Association of coronary calcium, carotid wall thickness, and carotid plaque progression with low-density lipoprotein and high-density lipoprotein particle concentration measured by ion mobility (from multiethnic study of atherosclerosis [mesa]). Am J Cardiol. 2021;142:52–58. doi:10.1016/j.amjcard.2020.11.026. 33278360 PMC7882028

[11] Peng J, Liu M, Liu H, et al. Lipoprotein (a)-mediated vascular calcification: population-based and in vitro studies. Metabolism. 2022;127:154960. doi:10.1016/j.metabol.2021.154960. 34954251

[12] Mccullough PA. Effect of lipid modification on progression of coronary calcification. J Am Soc Nephrol. 2005;16 Suppl 2(11_suppl_2):S115–S119. doi:10.1681/ASN.2005060664. 16251246

[13] Lee DY, Kim JH, Park SE, et al. Effects of low-density lipoprotein cholesterol on coronary artery calcification progression according to high-density lipoprotein cholesterol levels. Arch Med Res. 2017;48(3):284–291. doi:10.1016/j.arcmed.2017.06.005. 28923331

[14] Stompór T, Pasowicz M, Sulłowicz W, et al. An association between coronary artery calcification score, lipid profile, and selected markers of chronic inflammation in esrd patients treated with peritoneal dialysis. Am J Kidney Dis. 2003;41(1):203–211. doi:10.1053/ajkd.2003.50005. 12500238

[15] Shanahan CM, Crouthamel MH, Kapustin A, et al. Arterial calcification in chronic kidney disease: key roles for calcium and phosphate. Circ Res. 2011;109(6):697–711. doi:10.1161/CIRCRESAHA.110.234914. 21885837 PMC3249146

[16] Raggi P, Chertow GM, Torres PU, et al. The advance study: a randomized study to evaluate the effects of cinacalcet plus low-dose vitamin d on vascular calcification in patients on hemodialysis. Nephrol Dial Transplant. 2011;26(4):1327–1339. doi:10.1093/ndt/gfq725. 21148030

[17] Nishizawa Y, Mizuiri S, Yorioka N, et al. Determinants of coronary artery calcification in maintenance hemodialysis patients. J Artif Organs. 2015;18(3):251–256. doi:10.1007/s10047-015-0823-3. 25805429

[18] Zhang H, Xiang S, Dai Z, et al. Asymmetric dimethylarginine level as biomarkers of cardiovascular or all-cause mortality in patients with chronic kidney disease: a meta-analysis. Biomarkers. 2021;26(7):579–585. doi:10.1080/1354750X.2021.1954694. 34253095

[19] Pencak P, Czerwieńska B, Ficek R, et al. Calcification of coronary arteries and abdominal aorta in relation to traditional and novel risk factors of atherosclerosis in hemodialysis patients. BMC Nephrol. 2013;14(1):10. doi:10.1186/1471-2369-14-10. 23317172 PMC3556324

[20] Cui Y, Huang H, Ren W, et al. Parathyroidectomy is associated with reversed nondipping heart rate that impacts mortality in chronic kidney disease patients. Endocr Pract. 2022;28(2):148–158. doi:10.1016/j.eprac.2021.02.007. 33610808

[21] Wang X, Yuan L, Shi R, et al. Predictors of coronary artery calcification and its association with cardiovascular events in patients with chronic kidney disease. Ren Fail. 2021;43(1):1172–1179. doi:10.1080/0886022X.2021.1953529. 34315328 PMC8330733

[22] Bellasi A, Di Lullo L, Russo D, et al. Predictive value of measures of vascular calcification burden and progression for risk of death in incident to dialysis patients. J Clin Med. 2021;10(3):376. doi:10.3390/jcm10030376. 33498192 PMC7863918

[23] Hwang Z, Suh KJ, Chen D, et al. Imaging features of soft-tissue calcifications and related diseases: a systematic approach. Korean J Radiol. 2018;19(6):1147. doi:10.3348/kjr.2018.19.6.1147. 30386146 PMC6201973

[24] Xiong L, Chen Q, Cheng Y, et al. The relationship between coronary artery calcification and bone metabolic markers in maintenance hemodialysis patients. BMC Nephrol. 2023;24(1):238. doi:10.1186/s12882-023-03286-z. 37582785 PMC10428586

[25] Chen J, Budoff MJ, Reilly MP, et al. Coronary artery calcification and risk of cardiovascular disease and death among patients with chronic kidney disease. JAMA Cardiol. 2017;2(6):635–643. doi:10.1001/jamacardio.2017.0363. 28329057 PMC5798875

[26] Matsuoka M, Iseki K, Tamashiro M, et al. Impact of high coronary artery calcification score (cacs) on survival in patients on chronic hemodialysis. Clin Exp Nephrol. 2004;8(1):54–58. doi:10.1007/s10157-003-0260-0. 15067517

[27] Malluche HH, Blomquist G, Monier-Faugere M, et al. High parathyroid hormone level and osteoporosis predict progression of coronary artery calcification in patients on dialysis. J Am Soc Nephrol. 2015;26(10):2534–2544. doi:10.1681/ASN.2014070686. 25838468 PMC4587691

[28] Garland JS, Holden RM, Groome PA, et al. Prevalence and associations of coronary artery calcification in patients with stages 3 to 5 ckd without cardiovascular disease. Am J Kidney Dis. 2008;52(5):849–858. doi:10.1053/j.ajkd.2008.04.012. 18562059

[29] Ge Y, Wu B, Yu X, et al. Association of serum sclerostin level, coronary artery calcification, and patient outcomes in maintenance dialysis patients. Blood Purif. 2022;51(3):260–269. doi:10.1159/000516410. 34161949

[30] Han X, Cao Y, Ju Z, et al. Assessment of regional left ventricular myocardial strain in patients with left anterior descending coronary stenosis using computed tomography feature tracking. BMC Cardiovasc Disord. 2020;20(1):362. doi:10.1186/s12872-020-01644-5. 32770941 PMC7414558

[31] Blanke H, Cohen M, Schlueter GU, et al. Electrocardiographic and coronary arteriographic correlations during acute myocardial infarction. Am J Cardiol. 1984;54(3):249–255. doi:10.1016/0002-9149(84)90176-0. 6464999

[32] Cozzolino M, Ciceri P, Galassi A, et al. The key role of phosphate on vascular calcification. Toxins (Basel). 2019;11(4):213. doi:10.3390/toxins11040213. 30970562 PMC6521180

[33] Shimizu M, Fujii H, Kono K, et al. Clinical implication of consistently strict phosphate control for coronary and valvular calcification in incident patients undergoing hemodialysis. J Atheroscler Thromb. 2023;30(11):1568–1579. doi:10.5551/jat.64159. 36990726 PMC10627770

[34] Panh L, Ruidavets JB, Rousseau H, et al. Association between serum alkaline phosphatase and coronary artery calcification in a sample of primary cardiovascular prevention patients. Atherosclerosis. 2017;260:81–86. doi:10.1016/j.atherosclerosis.2017.03.030. 28371683

[35] Guo J, Zeng M, Zhang Y, et al. Serum alkaline phosphatase level predicts cardiac valve calcification in maintenance hemodialysis patients. Blood Purif. 2020;49(5):550–559. doi:10.1159/000505846. 32050204

[36] Ishimura E, Okuno S, Okazaki H, et al. Significant association between bone-specific alkaline phosphatase and vascular calcification of the hand arteries in male hemodialysis patients. Kidney Blood Press Res. 2014;39(4):299–307. doi:10.1159/000355807. 25300371

[37] Wu GY, Xu BD, Wu T, et al. Correlation between serum parathyroid hormone levels and coronary artery calcification in patients without renal failure. Biomed Rep. 2016;5(5):601–606. doi:10.3892/br.2016.761. 27882224 PMC5103683

[38] Hou Y, Liu W, Zheng C, et al. Role of vitamin d in uremic vascular calcification. Biomed Res Int. 2017;2017:2803579–2803513. doi:10.1155/2017/2803579. 28286758 PMC5329659

[39] Brahmbhatt S, Mikhail M, Islam S, et al. Vitamin d and abdominal aortic calcification in older african american women, the poda clinical trial. Nutrients. 2020;12(3):861. doi:10.3390/nu12030861. 32213826 PMC7146156

[40] Liu T, Zuo R, Wang J, et al. Association between serum 25-hydroxyvitamin d and abdominal aortic calcification: a large cross-sectional study. Int J Clin Pract. 2023;2023:1621873–1621810. doi:10.1155/2023/1621873. 36815008 PMC9940955

[41] Eddington H, Chinnadurai R, Alderson H, et al. A randomised controlled trial to examine the effects of cinacalcet on bone and cardiovascular parameters in haemodialysis patients with advanced secondary hyperparathyroidism. BMC Nephrol. 2021;22(1):106. doi:10.1186/s12882-021-02312-2. 33757437 PMC7989372

[42] Mediero A, Wilder T, Shah L, et al. Adenosine a(2a) receptor (a2ar) stimulation modulates expression of semaphorins 4d and 3a, regulators of bone homeostasis. Faseb J. 2018;32(7):3487–3501. doi:10.1096/fj.201700217R. 29394106 PMC5998975

[43] Wu Y, Zhang Q, Zhao B, et al. Effect and mechanism of propranolol on promoting osteogenic differentiation and early implant osseointegration. Int J Mol Med. 2021;48(4):1. doi:10.3892/ijmm.2021.5024. PMC8416142 34414453

[44] Kong Y, Liang Q, Chen Y, et al. Hyaluronan negatively regulates vascular calcification involving bmp2 signaling. Lab Invest. 2018;98(10):1320–1332. doi:10.1038/s41374-018-0076-x. 29785051

[45] Diederichsen SZ, Grønhøj MH, Mickley H, et al. Ct-detected growth of coronary artery calcification in asymptomatic middle-aged subjects and association with 15 biomarkers. JACC Cardiovasc Imaging. 2017;10(8):858–866. doi:10.1016/j.jcmg.2017.05.010. 28797406

[46] Cardoso R, Generoso G, Staniak HL, et al. Predictors of coronary artery calcium incidence and progression: the brazilian longitudinal study of adult health (elsa-brasil). Atherosclerosis. 2020;309:8–15. doi:10.1016/j.atherosclerosis.2020.07.003. 32858396

[47] Generoso G, Bensenor IM, Santos RD, et al. High-density lipoprotein-cholesterol subfractions and coronary artery calcium: the elsa-brasil study. Arch Med Res. 2019;50(6):362–367. doi:10.1016/j.arcmed.2019.10.006. 31678894

[48] Seyahi N, Cebi D, Altiparmak MR, et al. Progression of coronary artery calcification in renal transplant recipients. Nephrol Dial Transplant. 2012;27(5):2101–2107. doi:10.1093/ndt/gfr558. 21965591

[49] Xu C, Smith ER, Tiong MK, et al. Interventions to attenuate vascular calcification progression in chronic kidney disease: a systematic review of clinical trials. J Am Soc Nephrol. 2022;33(5):1011–1032. doi:10.1681/ASN.2021101327. 35232774 PMC9063901

[50] Wen W, Portales-Castillo I, Seethapathy R, et al. Intravenous sodium thiosulphate for vascular calcification of hemodialysis patients—a systematic review and meta-analysis. Nephrol Dial Transplant. 2023;38(3):733–745. doi:10.1093/ndt/gfac171. 35521751 PMC10111152

[51] Vermeulen EA, Vervloet MG. Magnesium administration in chronic kidney disease. Nutrients. 2023;15(3):547. doi:10.3390/nu15030547. 36771254 PMC9920010

[52] Pan W, Jie W, Huang H. Vascular calcification: molecular mechanisms and therapeutic interventions. MedComm (2020). 2023;4(1):e200. doi:10.1002/mco2.200. 36620697 PMC9811665

[53] Vaziri ND, Norris KC. Reasons for the lack of salutary effects of cholesterol-lowering interventions in end-stage renal disease populations. Blood Purif. 2013;35(1-3):31–36. doi:10.1159/000345176. 23343544 PMC3595172

[54] Chen Z, Qureshi AR, Parini P, et al. Does statins promote vascular calcification in chronic kidney disease? Eur J Clin Invest. 2017;47(2):137–148. doi:10.1111/eci.12718. 28036114

[55] Nakagami H, Osako MK, Morishita R. New concept of vascular calcification and metabolism. Curr Vasc Pharmacol. 2011;9(1):124–127. doi:10.2174/157016111793744742. 21044018

[56] Baigent C, Landray MJ, Reith C, et al. The effects of lowering LDL cholesterol with simvastatin plus ezetimibe in patients with chronic kidney disease (study of heart and renal protection): a randomised placebo-controlled trial. Lancet. 2011;377(9784):2181–2192. doi:10.1016/S0140-6736(11)60739-3. 21663949 PMC3145073

[57] Miyazaki Anzai S, Masuda M, Demos Davies KM, et al. Endoplasmic reticulum stress effector ccaat/enhancer-binding protein homologous protein (CHOP) regulates chronic kidney disease–induced vascular calcification. J Am Heart Assoc. 2014;3(3):e000949. doi:10.1161/JAHA.114.000949. 24963104 PMC4309099

[58] Tacey A, Qaradakhi T, Brennan-Speranza T, et al. Potential role for osteocalcin in the development of atherosclerosis and blood vessel disease. Nutrients. 2018;10(10):1426. doi:10.3390/nu10101426. 30287742 PMC6213520

[59] Kuzan A, Chwiłkowska A, Maksymowicz K, et al. Relationships between osteopontin, osteoprotegerin, and other extracellular matrix proteins in calcifying arteries. Biomedicines. 2024;12(4):847. doi:10.3390/biomedicines12040847. 38672202 PMC11048129

[60] Tang X, Qian H, Zeng M, et al. Predictive model for severe coronary artery calcification in ESKD patients. medRxiv [Preprint]. doi:10.1101/2023.12.16.23300066. PMC11232636 38874139

