# Supplementary material for: Predictive nomogram model for severe coronary artery calcification in end-stage kidney disease patients
Source: Ren Fail. 2024 Jun 14;46(2):2365393. doi: 10.1080/0886022X.2024.2365393 (PMC11232636; doi:10.1080/0886022X.2024.2365393)
Supplement: Supplemental Material [file IRNF_A_2365393_SM3326.zip › Supplemental figures.docx]

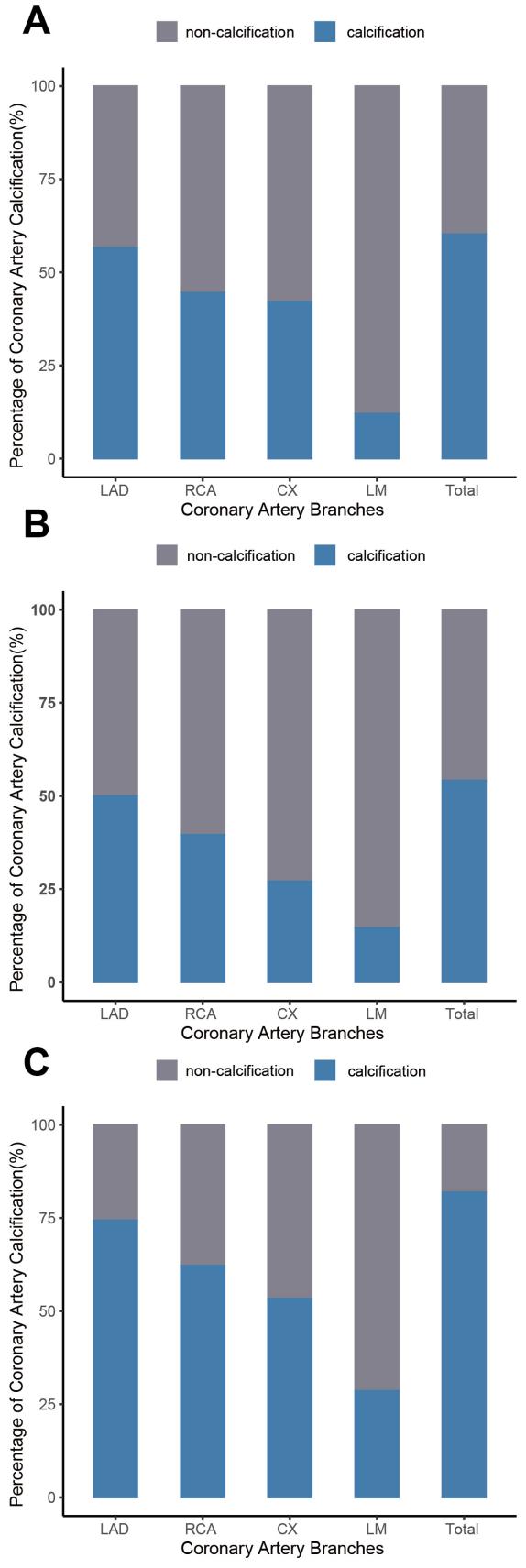


**Figure S1.Percentage of CAC in the four coronary branches**

(A) Predialysis patients. (B) Hemodialysis patients. (C)Peritoneal dialysis patients.

Abbreviations: LAD: left anterior descending branch; LM: left main trunk; CX: circumflex branch; RCA: right coronary artery; CAC: coronary artery calcification.
