# Supplementary material for: Predictive nomogram model for severe coronary artery calcification in end-stage kidney disease patients
Source: Ren Fail. 2024 Jun 14;46(2):2365393. doi: 10.1080/0886022X.2024.2365393 (PMC11232636; doi:10.1080/0886022X.2024.2365393)
Supplement: Supplemental Material [file IRNF_A_2365393_SM3326.zip › Supplemental tables.docx]

**Table S1. Comparison of Coronary Artery Branch Calcification in ESKD Patients with Different Dialysis Modalities**

| **Variables, n(%)** | **Predialysis**  **(n=44)** | **HD**  **(n= 237)** | **PD**  **(n=85)** | **Total**  **(n=369)** |
| --- | --- | --- | --- | --- |
| **LAD calcification** | 24(54.55) | 173(73.00) | 49(57.65) | 248(67.21) |
| **RCA calcification** | 19(43.18) | 146(61.6) | 38(44.71) | 204(55.28) |
| **CX calcification** | 13(29.55) | 125(52.74) | 35(41.18) | 175(47.43) |
| **LM calcification** | 7(15.90) | 68(28.69) | 10(11.76) | 85(23.04) |
| **Total calcification** | 26(59.9) | 191(80.59) | 52(61.18) | 271(73.44) |

Abbreviations: ESKD: end-stage kidney disease; LAD: left anterior descending branch; LM: left main trunk; CX: circumflex branch; RCA: right coronary artery; HD: hemodialysis; PD: peritoneal dialysis.

**Table S2. Clinical Characteristics and Laboratory Results of LAD Calcification in ESKD Patients**

| **Variables** | **LAD=0**  **（n=122）** | **0<LAD≤183.4**  **（n=121）** | **LAD>183.4**  **（n=126）** | ***P*** |
| --- | --- | --- | --- | --- |
| **Demographics** |  |  |  |  |
| Age (years) | 44.16±11.81 | 52.11±11.76 | 51.90±11.23 | ***<0.001^*^*** |
| Women,n (%) | 55.00(45.08) | 52.00(42.98) | 47.00(37.30) | 0.214 |
| BMI (kg/m^2^) | 21.38±3.40 | 23.16±3.82 | 23.00±3.95 | ***0.001^*^*** |
| SBP (mmHg) | 140.10±23.33 | 137.33±20.87 | 139.36±24.38 | 0.806 |
| DBP (mmHg) | 87.50±15.22 | 80.75±14.22 | 83.43±13.54 | ***0.029^*^*** |
| **Dialysis mode, n (%)** |  |  |  |  |
| Predialysis | 20.00(16.39) | 16.00(13.22) | 8.00(6.35) | ***0.014^*^*** |
| Hemodialysis | 61.00(50.00) | 77.00(63.64) | 99.00(78.57) | ***<0.001^*^*** |
| Peritoneal dialysis | 37.00(30.33) | 29.00(24.17) | 19.00(15.20) | ***0.005^*^*** |
| **Dialysis vintage(months)** | 60.00(3.88-97.75) | 66.00(20.00-108.00) | 96.00(51.00-132.75) | ***<0.001^*^*** |
| **Comorbidities, n (%)** |  |  |  |  |
| Diabetic mellitus | 12.00(9.84) | 24.00(19.83) | 23.00(18.25) | 0.073 |
| Hypertension | 87.00(71.31) | 95.00(78.51) | 105.00(83.33) | ***0.023^*^*** |
| **Cause of ESKD, n (%)** |  |  |  |  |
| CGN | 91.00(74.59) | 76.00(62.81) | 88.00(69.84) | 0.430 |
| DN | 9.00(7.38) | 16.00(13.22) | 13.00(10.32) | 0.455 |
| HN | 2.00(1.64) | 5.00(4.13) | 2.00(1.59) | 0.968 |
| Polycystic kidney disease | 5.00(4.10) | 6.00(5.00) | 7.00(5.60) | 0.586 |
| Other | 15.00(12.30) | 19.00(15.7) | 16.00(12.70) | 0.933 |
| **Medication history, n (%)** |  |  |  |  |
| Lipid-lowering treatment | 10.00(8.26) | 17.00(14.05) | 16.00(12.80) | 0.274 |
| Dihydropyridine CCBs | 62.00(51.24) | 62.00(51.24) | 70.00(56.00) | 0.454 |
| ACEI/ARB | 38.00(31.40) | 28.00(23.14) | 45.00(36.29) | 0.399 |
| β-receptor blocker | 45.00(37.19) | 47.00(38.84) | 61.00(48.80) | 0.064 |
| Phosphate binders | 49.00(40.50) | 41.00(33.88) | 56.00(44.80) | 0.482 |
| Active vitamin D sterols | 42.00(34.71) | 44.00(36.36) | 45.00(36.00) | 0.835 |
| Cinacalcet | 29.00(23.97) | 32.00(26.45) | 39.00(31.20) | 0.203 |
| **Laboratory values** |  |  |  |  |
| Hemoglobin (g/l) | 98.00±20.41 | 102.10±19.04 | 103.96±20.79 | ***0.020^*^*** |
| Hematocrit (%) | 30.15±6.51 | 31.79±6.11 | 32.48±6.48 | ***0.004^*^*** |
| Glucose (mmol/l) | 4.41±1.15 | 4.75±2.22 | 4.70±2.36 | 0.263 |
| Creatinine (μmol/l) | 906.40±318.97 | 897.84±307.34 | 877.73±288.69 | 0.458 |
| Urea (mmol/l) | 23.59±9.39 | 24.54±12.13 | 21.71±7.03 | 0.126 |
| TC (mmol/l) | 4.32±1.45 | 4.20±1.01 | 3.98±1.11 | ***0.024^*^*** |
| TG (mmol/l) | 1.75±1.26 | 1.63±1.01 | 1.84±1.39 | 0.547 |
| LDL-C (mmol/l) | 2.72±1.08 | 2.62±0.74 | 2.48±0.84 | ***0.034^*^*** |
| HDL-C (mmol/l) | 1.06±0.32 | 1.00±0.27 | 0.92±0.26 | ***<0.001^*^*** |
| Lpa (mmol/l) | 357.93±267.59 | 327.03±283.54 | 313.10±292.98 | 0.212 |
| Albumin (g/l) | 37.72±5.52 | 36.65±5.74 | 37.16±4.77 | 0.422 |
| Ca (mmol/l) | 2.30±0.25 | 2.33±0.28 | 2.43±0.24 | ***<0.001^*^*** |
| Adjusted Ca (mmol/l) | 2.35±0.23 | 2.40±0.24 | 2.49±0.22 | ***<0.001^*^*** |
| Phosphorus (mmol/l) | 1.98±0.45 | 2.05±0.52 | 2.16±0.55 | ***0.005^*^*** |
| Ca×P (mmol^2^/l^2^) | 4.65±1.17 | 4.95±1.40 | 5.39±1.50 | ***<0.001^*^*** |
| ALP (U/l) | 117.55(78.78-224) | 141.00(82.00-255.00) | 168.25(102.22-335.58) | 0.595 |
| Log(ALP) | 5.00±0.96 | 5.12±0.90 | 5.28±0.88 | ***0.016^*^*** |
| BAP (μg/l) | 27.26±28.32 | 31.81±26.56 | 44.30±38.73 | ***<0.001^*^*** |
| Log(BAP) | 2.86±1.09 | 3.17±0.75 | 3.39±0.92 | ***<0.001^*^*** |
| 25-OH-D (ng/dl) | 43.57±30.28 | 40.15±25.90 | 46.69±25.37 | 0.361 |
| iPTH (pg/ml) | 518.50(184.47-1440.33) | 856.40(239.30-1302.55) | 1203.90(358.65-1779.15) | ***0.005^*^*** |
| Log(iPTH) | 6.15±1.24 | 6.34±1.27 | 6.60±1.22 | ***0.004^*^*** |

Abbreviations: ESKD: end-stage kidney disease; BMI: body mass index; SBP: systolic blood pressure; DBP: diastolic blood pressure; CGN: chronic glomerulonephritis; DN: diabetic nephropathy; HN: hypertensive nephropathy; CCB: calcium channel blocker; ACEI/ARB: angiotensin-converting enzyme inhibitors/angiotensin II receptor blockers; TC: total cholesterol; TG: triglyceride; LDL-C: low-density lipoprotein cholesterol; HDL-C: high-density lipoprotein cholesterol; Lpa: lipoprotein a; Ca: calcium; P: phosphorus; ALP: alkaline phosphatase; BAP: bone-type alkaline phosphatase; 25-OH-D: 25 hydroxyvitamin D; iPTH: intact parathyroid hormone. *P*-values are obtained from comparisons among the three groups. An asterisk(*) indicates *P*<0.05. Data forms are expressed as mean±standard deviation or median (Q1-Q3), except where indicated.

**Table S3. Univariate Regression Analysis of Clinical Data and Severe CACS in Patients with ESKD**

| **Variables** | **LAD CACS** |  | **Total CACS** |  |
| --- | --- | --- | --- | --- |
|  | **OR (95%CI)** | ***P*** | **OR (95%CI)** | ***P*** |
| **Demographics** |  |  |  |  |
| Age (years) | 1.027 (1.008,1.045) | ***0.005^*^*** | 1.042 (1.023,1.062) | ***<0.001^*^*** |
| Women,n (%) | 0.756 (0.487,1.175) | 0.214 | 0.772 (0.499,1.195) | 0.246 |
| BMI (kg/m^2^ ) | 1.053 (0.993,1.116) | 0.086 | 1.027 (0.969,1.088) | 0.367 |
| SBP (mmHg) | 1.001 (0.992,1.011) | 0.800 | 0.999 (0.990,1.009) | 0.928 |
| DBP (mmHg) | 0.997 (0.982,1.012) | 0.656 | 0.988 (0.974,1.003) | 0.113 |
| **Dialysis mode, n (%)** |  |  |  |  |
| Predialysis | 0.390 (0.175,0.867) | ***0.021^*^*** | 0.370 (0.166,0.822) | ***0.015^*^*** |
| Hemodialysis | 2.790 (1.700,4.579) | ***<0.001^*^*** | 2.989 (1.823,4.903) | ***<0.001^*^*** |
| Peritoneal dialysis | 0.478 (0.272,0.840) | ***0.010^*^*** | 0.444 (0.253,0.779) | ***0.005^*^*** |
| **Dialysis vintage(months)** | 1.007 (1.003,1.011) | ***<0.001^*^*** | 1.008 (1.004,1.011) | ***<0.001^*^*** |
| **Comorbidities, n (%)** |  |  |  |  |
| Diabetic mellitus | 1.284 (0.723,2.280) | 0.393 | 1.320 (0.746,2.334) | 0.340 |
| Hypertension | 1.676 (0.966,2.908) | 0.066 | 2.276 (1.282,4.041) | ***0.005^*^*** |
| **Cause of ESKD, n (%)** |  |  |  |  |
| CGN | 1.054 (0.661,1.682) | 0.826 | 1.009 (0.635,1.603) | 0.969 |
| DN | 1.003 (0.494,2.036) | 0.993 | 1.081 (0.539,2.170) | 0.826 |
| HN | 0.544 (0.111,2.657) | 0.452 | 0.518 (0.106,2.530) | 0.416 |
| Polycystic kidney disease | 1.246 (0.471,3.297) | 0.658 | 1.527 (0.587,3.970) | 0.386 |
| Other | 0.894 (0.473,1.691) | 0.731 | 0.939 (0.501,1.761) | 0.845 |
| **Medication history, n (%)** |  |  |  |  |
| Lipid-lowering treatment | 1.169 (0.604,2.261) | 0.643 | 1.238 (0.644,2.377) | 0.522 |
| Dihydropyridine CCBs | 1.211 (0.785,1.869) | 0.387 | 1.323 (0.859,2.037) | 0.204 |
| ACEI/ARB | 1.519 (0.956,2.413) | 0.077 | 1.493 (0.943,2.365) | 0.088 |
| β-receptor blocker | 1.554 (1.005,2.404) | ***0.048^*^*** | 1.648 (1.068,2.543) | ***0.029^*^*** |
| Phosphate binders | 1.371 (0.884,2.125) | 0.159 | 1.697 (1.097,2.623) | ***0.017^*^*** |
| Active vitamin D sterols | 1.020 (0.651,1.601) | 0.930 | 0.808 (0.514,1.270) | 0.356 |
| Cinacalcet | 1.346 (0.835,2.168) | 0.222 | 1.414 (0.881,2.269) | 0.152 |
| **Laboratory values** |  |  |  |  |
| Hemoglobin (g/l) | 1.010 (0.999,1.021) | 0.078 | 1.009 (0.999,1.020) | 0.096 |
| Hematocrit (%) | 1.038 (1.003,1.074) | ***0.034^*^*** | 1.035 (1.001,1.071) | ***0.047^*^*** |
| Glucose (mmol/l) | 1.029 (0.926,1.143) | 0.595 | 1.031 (0.929,1.145) | 0.567 |
| Creatinine (μmol/l) | 0.999 (0.999,1.000) | 0.465 | 1.000 (0.999,1.001) | 0.745 |
| Urea (mmol/l) | 0.967 (0.943,0.996) | ***0.027^*^*** | 0.974 (0.948,1.000) | ***0.049^*^*** |
| TC (mmol/l) | 0.812 (0.671,0.984) | ***0.034^*^*** | 0.757 (0.622,0.921) | ***0.005^*^*** |
| TG (mmol/l) | 1.100 (0.928,1.303) | 0.273 | 1.012 (0.852,1.202) | 0.894 |
| LDL-C (mmol/l) | 0.775 (0.600,1.001) | 0.050 | 0.721 (0.556,0.935) | ***0.014^*^*** |
| HDL-C (mmol/l) | 0.219 (0.092,0.525) | ***<0.001^*^*** | 0.206 (0.086,0.493) | ***<0.001^*^*** |
| Lpa (mmol/l) | 0.999 (0.999,1.000) | 0.342 | 0.999 (0.999,1.000) | 0.127 |
| Albumin (g/l) | 0.999 (0.960,1.040) | 0.963 | 0.983 (0.944,1.023) | 0.390 |
| Ca (mmol/l) | 6.085 (2.491,14.864) | ***<0.001^*^*** | 4.806 (2.010,11.490) | ***<0.001^*^*** |
| Adjusted Ca (mmol/l) | 9.675 (3.458,27.070) | ***<0.001^*^*** | 8.714 (3.160,24.031) | ***<0.001^*^*** |
| Phosphorus (mmol/l) | 1.718 (1.125,2.626) | ***0.012^*^*** | 1.740 (1.141,2.653) | ***0.010^*^*** |
| Ca×P (mmol^2^/l^2^) | 1.361 (1.158,1.600) | ***<0.001^*^*** | 1.356 (1.155,1.592) | ***<0.001^*^*** |
| ALP (U/l) | 1.000 (1.000,1.001) | 0.699 | 1.000 (1.000,1.001) | 0.173 |
| Log(ALP) | 1.296 (1.027,1.636) | ***0.029^*^*** | 1.466 (1.159,1.853) | ***0.001^*^*** |
| BAP (μg/l) | 1.014 (1.005,1.022) | ***0.001^*^*** | 1.015 (1.007,1.023) | ***<0.001^*^*** |
| Log(BAP) | 1.602 (1.170,2.193) | ***0.003^*^*** | 1.688 (1.227,2.322) | ***0.001^*^*** |
| 25-OH-D (ng/dl) | 1.006 (0.999,1.014) | 0.113 | 1.002 (0.994,1.010) | 0.611 |
| iPTH (pg/ml) | 1.000 (1.000,1.001) | ***0.008^*^*** | 1.000 (1.000,1.001) | ***<0.001^*^*** |
| Log(iPTH) | 1.273 (1.058,1.532) | ***0.011^*^*** | 1.338 (1.110,1.613) | ***0.002^*^*** |

Abbreviations: ESKD: end-stage kidney disease; BMI: body mass index; SBP: systolic blood pressure; DBP: diastolic blood pressure; CGN: chronic glomerulonephritis; DN: diabetic nephropathy; HN: hypertensive nephropathy; CCB: calcium channel blocker; ACEI/ARB: angiotensin-converting enzyme inhibitors/angiotensin II receptor blockers; TC: total cholesterol; TG: triglyceride; LDL-C: low-density lipoprotein cholesterol; HDL-C: high-density lipoprotein cholesterol; Lpa: lipoprotein a; Ca: calcium; P: phosphorus; ALP: alkaline phosphatase; BAP: bone-type alkaline phosphatase; 25-OH-D: 25 hydroxyvitamin D; iPTH: intact parathyroid hormone. The *P*-values in the table are obtained through univariate logistic regression analysis for each index and calcification score. The asterisk （*） indicates *P*<0.05.

**Table S4.** **Clinical Characteristics and Laboratory Results of ESKD Patients Subgrouped by CACS in the Validation Cohort**

| **Variables** | **CACS=0**  **(n=22)** | **0<CACS≤400**  **(n= 51)** | **CACS>400**  **(n=54)** | ***P*** |
| --- | --- | --- | --- | --- |
| **Demographics** |  |  |  |  |
| Age (years) | 41.14±9.72 | 51.18±13.9 | 58.41±13.37 | ***<0.001^*^*** |
| Women,n (%) | 13(59.09) | 16(31.37) | 18(33.33) | 0.0868 |
| BMI (kg/m^2^ ) | 21.24±3.09 | 23.25±3.49 | 23.81±4.39 | ***0.0157^*^*** |
| SBP (mmHg) | 129.86±25.83 | 137.29±26.28 | 140.47±20.82 | 0.0953 |
| DBP (mmHg) | 78.91±15.27 | 85.16±17.03 | 81.68±12.31 | 0.7936 |
| **Dialysis mode, n (%)** |  |  |  |  |
| Predialysis | 2(9.09) | 8(15.69) | 2(3.7) | 0.2129 |
| Hemodialysis | 10(45.45) | 20(39.22) | 40(74.07) | ***0.0024^*^*** |
| Peritoneal dialysis | 9(42.86) | 23(45.1) | 11(20.75) | ***0.0202^*^*** |
| **Dialysis vintage(months)** | 48(15-120) | 48(9-96) | 72(30-120) | ***0.0405^*^*** |
| **Comorbidities, n (%)** |  |  |  |  |
| Diabetic mellitus | 1(4.55) | 14(28) | 10(18.52) | 0.4238 |
| Hypertension | 16(72.73) | 43(84.31) | 44(81.48) | 0.5296 |
| **Cause of ESKD, n (%)** |  |  |  |  |
| CGN | 5(22.73) | 6(11.76) | 14(25.93) | 0.4139 |
| DN | NA(NA) | 10(19.61) | 8(14.81) | 0.2314 |
| HN | 2(9.09) | 6(11.76) | 9(16.67) | 0.3370 |
| Polycystic kidney disease | NA(NA) | 2(3.92) | 1(1.85) | 0.8469 |
| Other | 15(68.18) | 30(58.82) | 24(44.44) | ***0.0414^*^*** |
| **Medication history, n (%)** |  |  |  |  |
| Lipid-lowering treatment | 2(9.09) | 15(29.41) | 15(27.78) | 0.1703 |
| Dihydropyridine CCBs | 7(31.82) | 25(49.02) | 29(53.7) | 0.1091 |
| ACEI/ARB | 10(45.45) | 18(35.29) | 24(44.44) | 0.8264 |
| β-receptor blocker | 8(36.36) | 20(39.22) | 29(53.7) | 0.1071 |
| Phosphate binders | 12(54.55) | 22(43.14) | 25(46.3) | 0.6530 |
| Active vitamin D sterols | 8(36.36) | 17(33.33) | 19(35.19) | 0.9826 |
| Cinacalcet | 7(31.82) | 9(17.65) | 10(18.52) | 0.2893 |
| **Laboratory values** |  |  |  |  |
| Hemoglobin (g/l) | 106.09±24.18 | 100.88±18.32 | 102.07±20.84 | 0.5719 |
| Hematocrit (%) | 33.14±7.54 | 31.91±5.7 | 33.73±9.1 | 0.5297 |
| Glucose (mmol/l) | 4.81±2.18 | 4.73±1.9 | 4.42±1.04 | 0.2795 |
| Creatinine (μmol/l) | 879.79±398.25 | 850.69±329.25 | 877.01±273.98 | 0.9210 |
| Urea (mmol/l) | 19.72±7.48 | 21.11±7.3 | 32.34±66.31 | 0.1737 |
| TC (mmol/l) | 4.24±1.01 | 4.04±1.17 | 4.03±1.28 | 0.5304 |
| TG (mmol/l) | 1.73±1.27 | 1.87±1.14 | 1.66±1.39 | 0.6756 |
| LDL-C (mmol/l) | 2.5±0.68 | 2.5±0.78 | 2.42±0.81 | 0.6105 |
| HDL-C (mmol/l) | 1.14±0.34 | 0.91±0.29 | 0.96±0.35 | 0.1018 |
| Lpa (mmol/l) | 201.39±208.62 | 313.34±297.11 | 299.66±314.85 | 0.2945 |
| Albumin (g/l) | 37.25±3.8 | 34.65±6.25 | 36.99±5.24 | 0.6266 |
| Ca (mmol/l) | 2.33±0.25 | 2.19±0.32 | 2.32±0.28 | 0.5339 |
| Adjusted Ca (mmol/l) | 2.38±0.26 | 2.3±0.31 | 2.39±0.26 | 0.6201 |
| Phosphorus (mmol/l) | 2±0.62 | 1.85±0.6 | 2.16±0.6 | 0.1030 |
| Ca×P (mmol^2^/l^2^) | 4.78±1.61 | 4.25±1.44 | 5.17±1.48 | 0.0750 |
| ALP (U/l) | 99(72-186.5) | 99(74.25-184.75) | 98.5(67.25-136.25) | 0.4378 |
| Log(ALP) | 4.85±0.88 | 4.95±1.04 | 4.77±0.76 | 0.5730 |
| BAP (μg/l) | 59.24±35.27 | 30.72±37.56 | 26.23±20.27 | ***0.0227^*^*** |
| Log(BAP) | 3.92±0.62 | 3±0.85 | 3.03±0.68 | ***0.0365^*^*** |
| 25-OH-D (ng/dl) | 272.85±1078.77 | 48.9±101.18 | 37.9±24.53 | 0.0852 |
| iPTH (pg/ml) | 410.65(70.5-858.78) | 245.9(109.1-513.45) | 276.3(96.2-743.9) | 0.9329 |
| Log(iPTH) | 5.55±1.46 | 5.41±1.61 | 5.38±1.75 | 0.7086 |

Abbreviations: ESKD: end-stage kidney disease; BMI: body mass index; SBP: systolic blood pressure; DBP: diastolic blood pressure; CGN: chronic glomerulonephritis; DN: diabetic nephropathy; HN: hypertensive nephropathy; CCB: calcium channel blocker; ACEI/ARB: angiotensin-converting enzyme inhibitors/angiotensin II receptor blockers; TC: total cholesterol; TG: triglyceride; LDL-C: low-density lipoprotein cholesterol; HDL-C: high-density lipoprotein cholesterol; Lpa: lipoprotein a; Ca: calcium; P: phosphorus; ALP: alkaline phosphatase; BAP: bone-type alkaline phosphatase; 25-OH-D: 25 hydroxyvitamin D; iPTH: intact parathyroid hormone. The *P*-values in the table are obtained through univariate logistic regression analysis of each index and calcification score. An asterisk (*) indicates a *P-*value less than 0.05.
